# Three-dimensional rotation electron diffraction: software *RED* for automated data collection and data processing

**DOI:** 10.1107/S0021889813027714

**Published:** 2013-11-15

**Authors:** Wei Wan, Junliang Sun, Jie Su, Sven Hovmöller, Xiaodong Zou

**Affiliations:** a Inorganic and Structural Chemistry and Berzelii Centre EXSELENT on Porous Materials, Department of Materials and Environmental Chemistry, Stockholm University, Arrhenius Laboratory, Stockholm SE-106 91, Sweden

**Keywords:** rotation electron diffraction, electron diffraction tomography, three-dimensional electron diffraction, structure analysis, electron diffraction, computer programs

## Abstract

Implementation of a computer program package for automated collection and processing of rotation electron diffraction (RED) data is described. The software package contains two computer programs: *RED* data collection and *RED* data processing. The *RED* data collection program controls the transmission electron microscope and the camera. Electron beam tilts at a fine step (0.05–0.20°) are combined with goniometer tilts at a coarse step (2.0–3.0°) around a common tilt axis, which allows a fine relative tilt to be achieved between the electron beam and the crystal in a large tilt range. An electron diffraction (ED) frame is collected at each combination of beam tilt and goniometer tilt. The *RED* data processing program processes three-dimensional ED data generated by the *RED* data collection program or by other approaches. It includes shift correction of the ED frames, peak hunting for diffraction spots in individual ED frames and identification of these diffraction spots as reflections in three dimensions. Unit-cell parameters are determined from the positions of reflections in three-dimensional reciprocal space. All reflections are indexed, and finally a list with *hkl* indices and intensities is output. The data processing program also includes a visualizer to view and analyse three-dimensional reciprocal lattices reconstructed from the ED frames. Details of the implementation are described. Data collection and data processing with the software *RED* are demonstrated using a calcined zeolite sample, silicalite-1. The structure of the calcined silicalite-1, with 72 unique atoms, could be solved from the RED data by routine direct methods.

## Abstract 2

Implementation of the *RED* software package for automated collection and processing of rotation electron diffraction data is described.

## 1. Introduction   

Single-crystal diffraction can be achieved using X-rays, neutrons or electrons. While the first two have always been both quantitative and three dimensional, electron diffraction (ED) was until very recently mainly done in two dimensions. Structure determination by electron crystallography has not been a conventional method for several reasons. Until recently, for three-dimensional structure determination, it was necessary to collect a small number of electron diffraction patterns manually (often on photographic film), each one very accurately aligned along a zone axis ([100], [110], [111], [120] *etc.*). This was very time consuming work and could only be done by highly trained and skilled persons. Although very complicated structures could be determined in this way, such as ν-AlFeCr (Zou *et al.*, 2003 ▶) with 129 unique atoms, it took months if not years to collect, process and analyse such data. This made the technique inferior to X-ray and neutron diffraction, which for decades have been done automatically in hours or days. With the advent of CCD cameras and computer-controlled electron microscopes, the scene has changed. It is now possible to collect thousands of ED frames, more or less automatically, on a modern transmission electron microscope, in about one hour.

There are currently two methods used for collecting three-dimensional electron diffraction data: automated diffraction tomography (ADT), developed by Kolb’s group in Mainz (Kolb *et al.*, 2007 ▶, 2008 ▶, 2011 ▶), and the rotation electron diffraction (RED) method, developed by Hovmöller and Zou’s group in Stockholm (Hovmöller, 2008 ▶; Zhang *et al.*, 2010 ▶). The principles of ADT have been described in a series of publications (Kolb *et al.*, 2007 ▶, 2008 ▶, 2011 ▶). ADT uses discrete steps of goniometer tilt (typically 1° steps) to cover a large portion of reciprocal space (Kolb *et al.*, 2010 ▶; Gorelik *et al.*, 2011 ▶, 2012 ▶), which is often combined with a continuous precession to fill in the space between the discrete goniometer tilt positions. When combined with precession, the geometry of ADT corresponds to the screenless precession method used for X-ray diffraction (Xuong *et al.*, 1968 ▶; Xuong & Freer, 1971 ▶). ADT uses specialized hardware for precession and scanning transmission electron microscopy for crystal tracking (Kolb *et al.*, 2011 ▶). RED combines discrete goniometer tilt steps (typically 2–3°) with very fine steps of beam tilt, typically 0.05–0.20°. The geometry of the RED method resembles the rotation method used for single-crystal X-ray diffraction (Arndt & Wonacott, 1977 ▶; Dauter, 1999 ▶). The geometry of the rotation method is simpler than the precession method, which may facilitate data processing and analysis. One important advantage of the RED method is that data collection can be controlled entirely by software and performed on a conventional transmission electron microscope without any additional hardware. The method works for both selected area electron diffraction (SAED) and nano-diffraction. Both ADT and RED methods are being used as routine techniques in an increasing number of laboratories. More than 50 structures, including both known and unknown ones, have already been solved by the RED method (Martínez-Franco *et al.*, 2013 ▶; Su *et al.*, 2013 ▶; Willhammar *et al.*, 2013 ▶; Zhang *et al.*, 2013 ▶). Today it has become routine work to collect essentially complete three-dimensional electron diffraction data from crystals as small as tens of nanometres, several orders of magnitude smaller than what can be studied on a synchrotron light source. Since there is no need to align the crystal, data collection is made very simple and fast.

We have previously reported the RED method for data collection (Zhang *et al.*, 2010 ▶), where the transmission electron microscope, crystal tilt and CCD camera were controlled by scripts written in Gatan’s *DigitalMicrograph* (http://www.gatan.com/imaging/dig_micrograph.php). Here we present the *RED* data collection software, which controls the transmission electron microscope and the camera. We also present the *RED* data processing program, which processes the three-dimensional electron diffraction data generated from the *RED* data collection program or by other methods. Finally we use a calcined zeolite sample, silicalite-1, as an example to illustrate the procedure of collecting and processing a three-dimensional electron diffraction data set by RED and the structure solution of silicalite-1 from RED data. Silicalite-1 is one of the most complex polymorphs of SiO_2_. It has an MFI zeolite framework type and contains a three-dimensional pore system (Flanigen *et al.*, 1978 ▶) that is interesting for separation and selective catalysis.

## 2. *RED* data collection   

**Figure 1:**
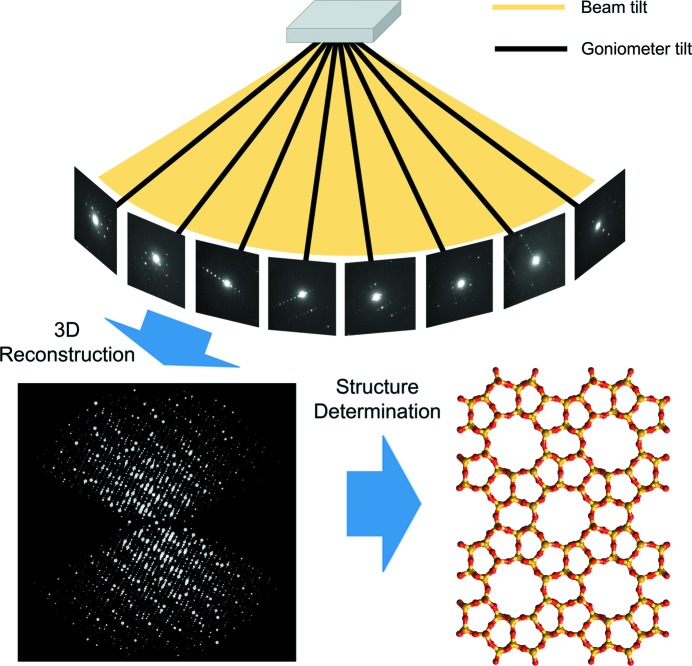
Schematic representation of the concept of the rotation electron diffraction (RED) method. RED data (individual ED frames) are collected by combining beam tilt and goniometer tilt. After three-dimensional reconstruction, the intensities and positions of the reflections in reciprocal space are obtained and can be visualized. After further data processing the unit cell is determined and the reflections are indexed. A resultant *hkl* list is then used to determine the atomic structure of the crystals. The example used here is the zeolite silicalite-1.

The concept of the RED method is illustrated in Fig. 1 ▶. Electron beam tilts in a fine step (0.05–0.20°) are combined with goniometer tilts in a coarse step (2.0–3.0°) around a common tilt axis, which allows a fine relative tilt to be achieved between the electron beam and crystal in a large tilt range. ED frames are collected at each combination of the beam tilt and goniometer tilt. Each ED frame is an intersection of the Ewald sphere with reciprocal space. The RED data collection is done by repeating the following two steps: (1) collect ED frames using a beam tilt in the range of ±α (α ≃ 1.0–2.0°) with a step of 0.05–0.20° and (2) rotate the crystal about the tilt axis by 2α.

In principle, RED data can be collected by keeping the beam fixed and tilting only the crystal using a goniometer on the microscope. In practice, however, because goniometer tilt is achieved by mechanical gears, the smallest step of tilting is limited to about 0.1°. When a goniometer is used at its minimum tilt step, it usually does not perform tilting smoothly. For example, it may stay stationary in some of the steps or jump a larger step in some others. On the other hand, beam tilt in a microscope is controlled by the electric current in the beam tilt deflectors. These can be changed in very fine and precise steps, enabling beam tilt control with a step down to below 0.001°. The limitation of beam tilt is that the maximum reliable (linear) tilt is usually within 3°. Close to the limit, the beam tilt may not be precise, and the deflectors for de-scan (compensation for the image and diffraction shift due to beam tilt) may not be able to keep the image or electron diffraction stationary. In practice, owing to the low precision of the goniometer tilt, the beam tilt range is usually set to ensure that there are overlapping frames between neighbouring beam tilt series. For example, if a set of 21 ED frames is recorded with 0.10° beam tilt step (from −1.00 to +1.00°) at each goniometer tilt of 2.0°, the last ED frame in one such set of 21 ED frames will be identical to the first ED frame of the next set after the goniometer tilt.

**Figure 2:**
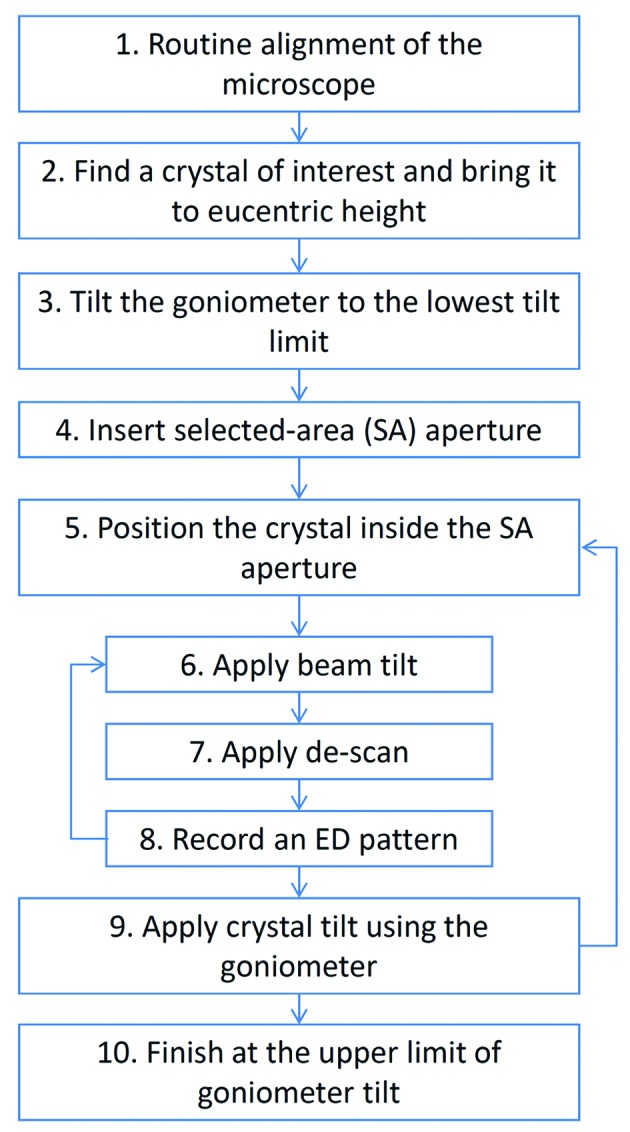
A flowchart showing the steps of a complete RED data collection procedure using the SAED mode and controlled by the *RED* data collection program.

The *RED* data collection program is used for automated collection of three-dimensional ED data by controlling beam scan and goniometer tilt, as well as recording ED frames at each given combination of beam tilt and goniometer tilt. A previous implementation was made using Gatan *Digital­Micro­graph* scripting, and the technical details and procedures were described in an earlier paper (Zhang *et al.*, 2010 ▶). A new implementation is now made, using C++. This is a standalone program that runs outside *DigitalMicrograph* (see supporting information for technical details^1^). The new design follows a similar procedure in data collection to the previous one (Zhang *et al.*, 2010 ▶), as shown in Fig. 2 ▶, but includes richer and improved functions. The calibration procedure for determining the relative strength of the beam tilt and image shift deflectors has been re-designed (see Appendix *A* [App appa] for a detailed description). The *RED* data collection program works on Windows only, as it needs to interface with the camera and microscope software which run only on Windows.

**Figure 3:**
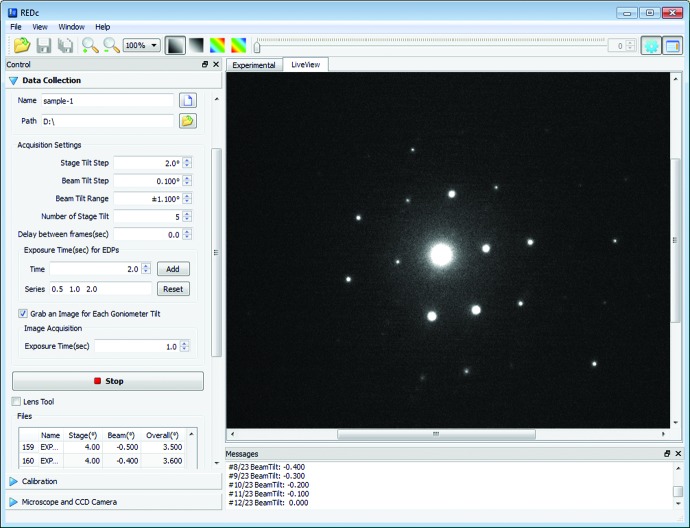
Graphical user interface of the *RED* data collection program.

The graphical user interface of the program is shown in Fig. 3 ▶. The beam and goniometer stage tilt steps, the beam tilt range and the exposure time can be defined by the user (Fig. S1a). The actual tilt angles of the goniometer often differ slightly from the intended tilt angle. The *RED* data collection program reads the actual goniometer tilt angles from the transmission electron microscope. The beam tilt and actual crystal tilt angles are saved automatically together with the ED frames. It is possible to collect several series of RED data with different exposure times, for example 0.5, 1.0 and 2.0 s, in one data collection run. This is helpful when there are intensity overflows. Strong reflections can be extracted from the ED frames with shorter exposure time, while weak reflections can be extracted from the ED frames with longer exposure time. The *RED* data collection program can run in either SAED or nano-diffraction mode. For SAED mode, the program includes a function for automatically switching the transmission electron microscope between image and diffraction mode, making it easy to track the crystal and compensate for drift if necessary. For nano-diffraction mode, crystal tracking is done in diffraction mode with defocused ED frames, where the crystal image in the direct beam is used. After crystal tracking the diffraction focus is set to a fixed value which will be used throughout the data collection. Images of the crystal at each goniometer tilt angle can be automatically saved and used for electron tomography to reconstruct the crystal morphology. Together with the reconstructed reciprocal lattice, it is possible to index different crystal faces. The crystal size and morphology may be useful for correcting absorption and dynamical effects.

## 3. *RED* data processing   

In order to obtain a complete data set, *RED* usually runs in a large tilt range with fine tilt steps, for example ±70° with a step of 0.10°. A RED data set like this contains over 1400 ED frames. It is not practical to process these ED frames one by one manually to extract information about the crystal. Instead a computer program must be used. *RED* data processing is specifically designed to process ED data collected in the manner described above, for example, those from the *RED* data collection program. However, the *RED* data processing program is not limited to processing of data from the *RED* data collection program. Any electron diffraction data collected by rotating the crystal or the beam can be processed, regardless of whether this is done manually or automatically using software.

**Figure 4:**
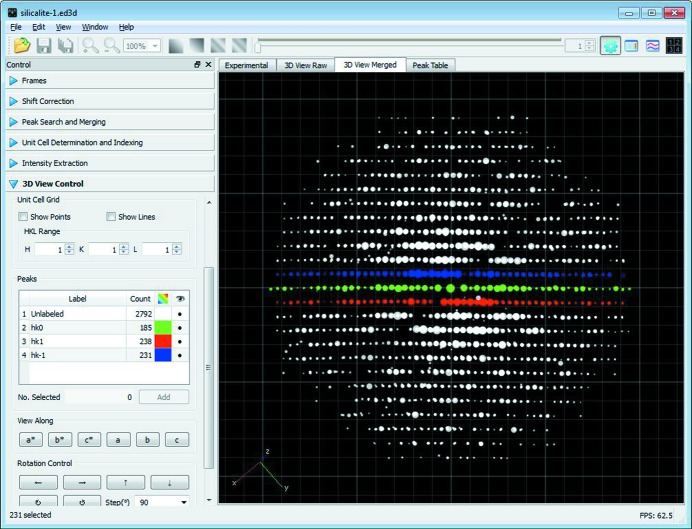
Graphical user interface of the *RED* data processing program. It shows the reconstructed three-dimensional reciprocal lattice for calcined silicalite-1, obtained from 1472 individual ED frames. The *hk*0, *hk*1 and *hk* 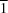 layers are marked in green, red and blue, respectively.

**Figure 5:**
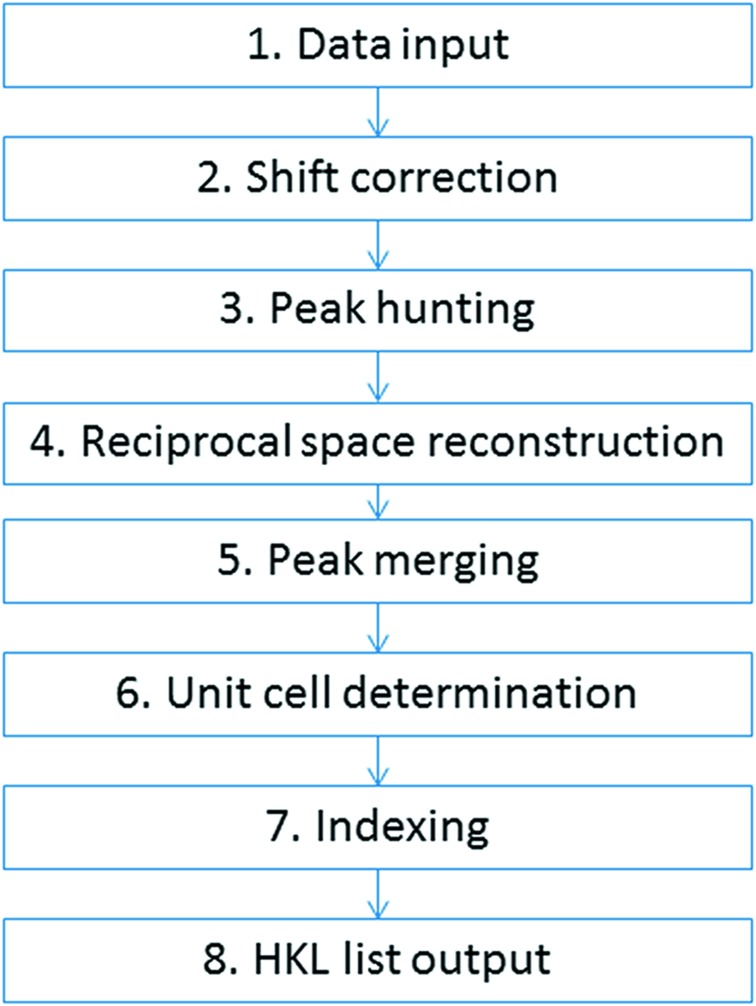
A flowchart showing the steps of data processing using the *RED* data processing program.

The *RED* data processing program processes the ED data collected by the data collection program and extracts useful information, for example, unit-cell parameters and diffraction intensities. These can be used as single-crystal diffraction data for *ab initio* structure solution and refinement. Raw RED data typically are composed of many hundreds of ED frames, which are intersections of the reciprocal lattice of the crystal with the Ewald sphere. Data processing involves reconstructing the three-dimensional reciprocal lattice from these two-dimensional ED frames, identifying reflections in three dimensions, determining the unit cell, indexing the reflections and extracting intensities. The *RED* data processing program runs under both Windows and Linux, and its graphical user interface under Windows is shown in Fig. 4 ▶. The flowchart of a full data processing sequence is given in Fig. 5 ▶. Each of the steps will be described in detail below.

### 3.1. ED frame and data input   

A RED data set collected using the *RED* data collection program contains an information file in plain text format and the raw data files of ED frames in MRC format (Crowther *et al.*, 1996 ▶). The information file is automatically saved during *RED* data collection. It includes electron wavelength, orientation of the tilt axis, reciprocal space sampling (number of pixels per reciprocal Å, denoted by pixel*Å), file names of the individual frames, and corresponding goniometer tilt, beam tilt and combined tilt. Here it acts as the input file for the data processing program. For three-dimensional ED frames collected by methods other than the *RED* software, it is necessary to prepare a separate information file.

### 3.2. Shift correction   

**Figure 6:**
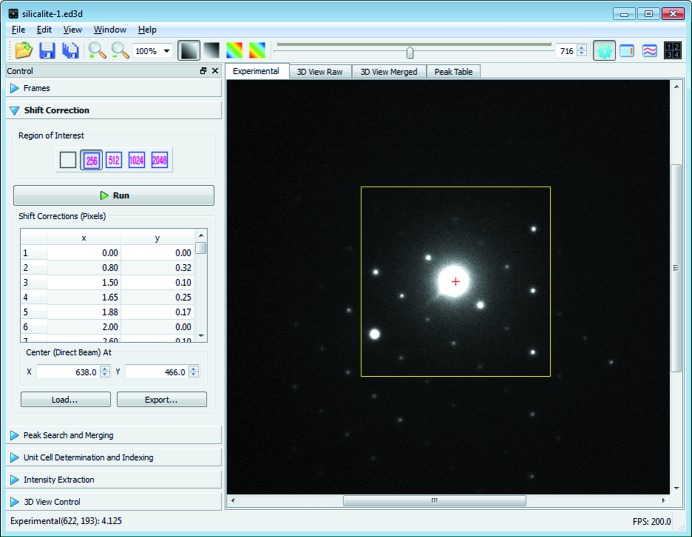
Shift correction window of the *RED* data processing program. An area of 256 × 256 pixels covering the direct beam is selected for cross-correlation calculation.

It is commonly observed that the direct beam (and with it the whole diffraction pattern) slightly shifts its position during data collection. The shift of the direct beam through the data set is found individually for each ED frame using cross correlation of a small user-defined area surrounding the direct beam (Fig. 6 ▶). The shift is usually within a few pixels and is corrected for each frame to sub-pixel accuracy in data processing by *RED*. The final data quality is not affected by this shift. To optimize the accuracy of the overall alignment of the direct beam, cross correlation is carried out between a frame and the summation of all previous frames after their corresponding shifts have been corrected for. This scheme is adopted from an image processing method developed for high-resolution transmission electron microscopy (TEM) micrographs (Wan *et al.*, 2012 ▶). As features of strong contrast dominate cross correlation, it is important that the direct beam is always the strongest in the user-defined area in each ED frame. Otherwise cross correlation may give erroneous results.

### 3.3. Peak hunting   

Peak hunting is a process to identify diffraction spots in the ED frames. These will be used later for reciprocal space reconstruction and unit-cell determination. The intensities of the diffraction spots will be extracted for structure solution. It is important that all reflections, both strong and weak, in high and low background, are picked up and noise peaks are rejected. This is a common blob detection problem in the field of computer vision (Lowe, 2004 ▶). ED frames contain spots with both intensities and background varying in large ranges. We tested various spot identification algorithms and did not find any one that is satisfactory for the ED data. Thus, we developed the following algorithm for identifying diffraction spots in ED frames.

**Figure 7:**
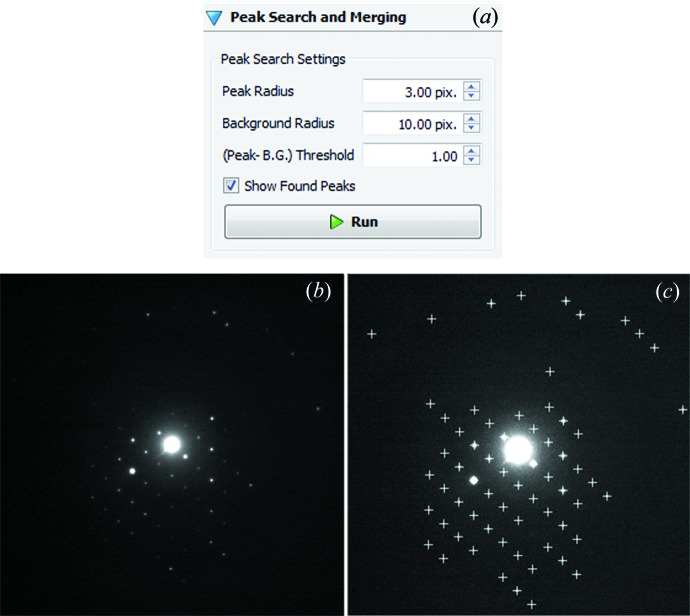
Peak hunting settings in the *RED* data processing program (*a*) and an example of peak hunting results (*c*) from an ED frame in the silicalite-1 data set (*b*). All visible peaks in (*b*) are detected by *RED* and marked with crosses in (*c*). The peak and background radii (*i.e*. standard deviations of Gaussian functions) as well as the peak–background threshold can be defined by the user.

The peak hunting algorithm adopted in *RED* data processing is as follows. In a first step, the original ED frame is convoluted with a Gaussian function with a relatively large standard deviation, typically more than ten pixels. The resulting frame has strongly blurred contrast and is used as a background frame. In a second step, the original ED frame is convoluted with another Gaussian function with a relatively small standard deviation, typically 0.5–2.0 pixels. The contrast of the original ED frame will be more or less preserved, but noise is reduced. The resultant frame is used as the diffraction frame. In a third step, the background frame from step 1 and the diffraction frame from step 2 are compared pixel by pixel. If intensities in the diffraction frame are higher than those at corresponding positions in the background frame by a certain user-defined threshold, the pixels are considered to belong to a diffraction spot. Finally, groups formed by pixels close to each other in each ED frame are considered as single diffraction spots. The position of the pixel with the highest intensity in each such group is taken as the final position of the diffraction spot (Fig. 7 ▶). The position of each diffraction spot is stored as *x*, *y* and frame number, where *x* and *y* are the coordinates of the diffraction spot on the two-dimensional ED frame.

### 3.4. Peak intensity extraction   

The intensity of each diffraction spot is evaluated after the position of the diffraction spot is determined. If the exposure time and beam intensity are controlled so that there is no overflow in the CCD frame (except probably for the direct beam), all diffraction spots will have essentially the same shape but different heights in intensity. The intensities of the diffraction spots can then be extracted by taking the pixel with the highest count or by integrating all pixels of each reflection. Accurate intensity evaluation from ED patterns has been studied previously (Zou *et al.*, 1993*a* ▶,*b* ▶).

In the *RED* data processing program, two methods for extracting intensities of the diffraction spots in the ED frames are included. One is to extract intensities from the Gaussian smoothed diffraction frames at the final positions of the diffraction spots as mentioned above. The intensities extracted are weighted averaged intensities of the pixels on and around the final positions of the diffraction spots (for smoothing using a Gaussian function with a standard deviation of one pixel, this is close to 64% of the central pixel plus 9% of the nearest four pixels). Another option for intensity extraction is through simple two-dimensional integration. A circular window slightly larger (defined by the user) than the diffraction spots is used to mask the diffraction spots. The circular window is enlarged by a few pixels and the average intensity of the area between the two circles is used as the background for the diffraction spot. The intensity of each pixel in the inner circle is reduced by the average background level. The sum of the background-removed intensities of all pixels in the inner circle is then taken as the final intensity of the diffraction spot.

### 3.5. Reciprocal space reconstruction and visualization   

**Figure 8:**
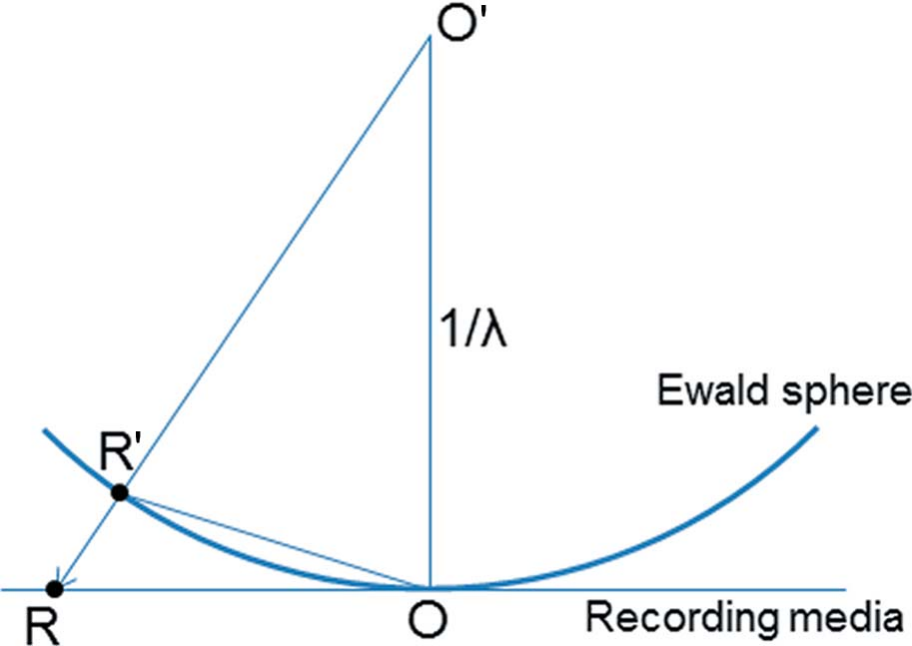
Back projection of a diffraction spot *R* in a diffraction pattern onto the Ewald sphere at *R*′. *O* is the centre of the diffraction pattern and *O*′ the centre of the Ewald sphere.

In order to reconstruct the three-dimensional reciprocal space from two-dimensional ED frames, one needs to correct for the effect of the Ewald sphere. Peak hunting gives the two-dimensional positions of many diffraction spots in each ED frame, as given by 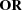 in Fig. 8 ▶. The two-dimensional diffraction spot is then back projected onto the Ewald sphere to recover the position where the reflection intersects the Ewald sphere, 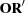. 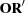 is then rotated around the tilt axis according to the tilt angle of the corresponding ED frame to obtain the final three-dimensional position of the diffraction spot in reciprocal space. This operation is performed on all diffraction spots found in the frames, and the result is a reconstructed three-dimensional reciprocal space which can be visualized by the *RED* program. The user can use the visualizer to examine the three-dimensional diffraction spots and make two-dimensional cuts of different layers of the reciprocal lattice.

It is possible to view the three-dimensional reciprocal lattice along any direction, including the main unit-cell axes. Groups of reflections can be marked in different colours and selected to be displayed, as shown in Fig. 4 ▶. Two-dimensional slices can be cut perpendicular to any arbitrary directions. Visual inspection of the two-dimensional slices *hk*0, *h*0*l*, 0*kl etc.* is very useful for space-group determination. Reflection statistics for finding possible systematic absences are also given by *RED* after the unit cell has been found and the reflections have been indexed. It is possible to cut sections such as *h*1*l*, *h*2*l*, *hk*3 and so on, *i.e.* sections that do not pass through reflection 000. This is especially interesting, since such sections cannot be obtained using conventional techniques for electron diffraction, such as SAED and precession.

### 3.6. Refinement of the orientation of the tilt axis   

**Figure 9:**
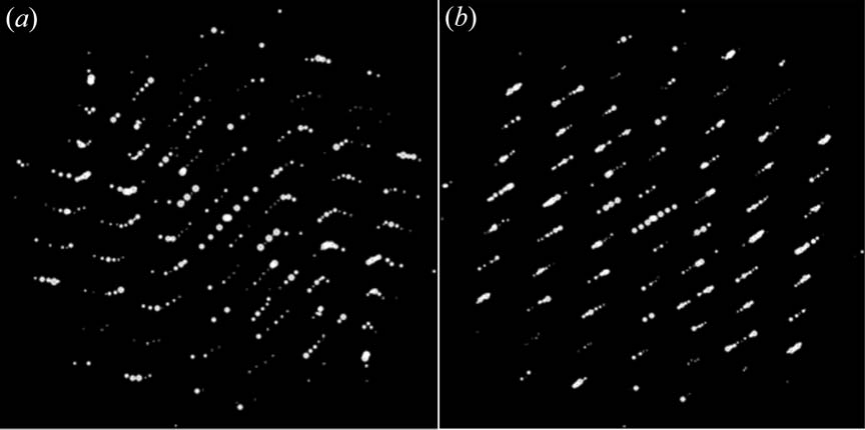
Reconstructed three-dimensional reciprocal lattice of silicalite-1 with (*a*) incorrectly specified tilt axis orientation (42.5°) and (*b*) correct tilt axis orientation (48.5°). Notice that the reflection rows are bent when the tilt axis orientation is wrong, as shown in (*a*). This can be used to refine the tilt axis orientation.

The orientation of the axis around which the crystal is rotated has to be pre-determined because the beam needs to be set to scan perpendicular to that axis during data collection. This information is also needed when reconstructing three-dimensional reciprocal space from ED frames in RED data. The tilt axis can be determined by tilting a crystal and following the centre of the Laue circle (Zhang *et al.*, 2010 ▶). The accuracy of the determination can later be improved during data processing. In ADT, the orientation of the tilt axis is determined by checking the sharpness of the cylindrical projection of reciprocal space assuming different orientations (Kolb *et al.*, 2009 ▶). A similar idea is adopted in RED to find the initial tilt axis. A data set needs to be collected from a well diffracting crystal (generating high-resolution data) with goniometer tilt only. Reciprocal space is first reconstructed using a random trial tilt axis orientation and visualized. If the trial tilt axis is off the true one by more than a few degrees, the reconstructed reflection lattices will appear bent, as shown in Fig. 9 ▶(*a*). The orientation is then changed in small steps and the resulting reciprocal lattice is visualized and examined. The tilt axis orientation is found when the reflection lattices are straight (Fig. 9 ▶ *b*). In our experience, the orientation can be found with a precision of about 1.0° using this method. The tilt axis orientation does not change over time for a certain microscope in the same configuration. Once found, it can be used for future reconstructions. The tilt axis in the ED frames may rotate by a few degrees owing to the rotation of ED patterns when the camera length or diffraction focus is changed. In principle, the above procedure should be performed at each camera length and focus configuration. In practice, for SAED the variation of diffraction focus between different data sets is usually small and there is no noticeable rotation of the ED patterns. It thus suffices to calibrate the rotation at different camera lengths. For nano-beam diffraction more dramatic changes in the diffraction focus may be possible. In such cases, the rotation can be calibrated at a few diffraction foci (preferably at different camera lengths) and interpolated for any focus used in data collection.

An error in the orientation of the tilt axis results in errors not only in the reconstructed reflection positions, *i.e.* bent reflection lattices, but also in unit-cell determination since the positions of the reflections are used (see below). Experiments show that, using RED data, unit-cell parameters with good accuracy (typically better than 1% in unit-cell lengths and angles) are obtained (Su *et al.*, 2013 ▶). Even if the tilt axis orientation is off by as much as ±2°, the unit-cell dimensions remain almost the same. Bent reflection lattices may also pose problems in indexing, as the reflections do not follow the reciprocal basis vectors perfectly. In *RED* data processing, the bending of the lattice is taken into consideration in indexing, and correct indices of the reflection can be found in spite of some bending, as will be discussed later.

### 3.7. Peak merging and intensity integration   

As the tilt step is usually small, most reflections will intersect with the Ewald sphere on several consecutive ED frames. After such diffraction spots are transformed into three dimensions, they will be close to each other, forming a group that belongs to one and the same reflection. In the peak merging step, the program will identify such groups of diffraction spots and merge them into one reflection. The program uses the distances between the diffraction spots in two-dimensional ED frames and also in three-dimensional reciprocal space to determine whether the diffraction spots belong to the same reflection, and uses the distances between groups of diffraction spots to separate the reflections.

After a group of diffraction spots have been merged into one reflection, its intensity and three-dimensional position will be determined. The final three-dimensional position of the reflection will be the intensity-weighted averaged position of all positions of the diffraction spots that belong to the reflection. There are two options to determine the final intensity. If the sampling is fine enough (typically ∼0.1° tilt steps) all reflections are present on several consecutive ED frames. The user can chose between picking the intensity from the diffraction frame with the highest value (default) or a three-dimensional integration over diffraction frames. The three-dimensional integration is done by integrating the intensities of the diffraction spots over the reciprocal difference vectors between the neighbouring spots:
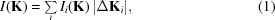
where *I*(**K**) is the integrated intensity for reflection **K**. *I_i_*(**K**) is the intensity of the diffraction spot on frame *i* and Δ**K** *_i_* = **K** *_i_* − **K**_*i*−1_ is the difference of the three-dimensional positions of the diffraction spots from frames *i* and *i*− 1. 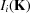 can be either the maximum value of the diffraction spot or the two-dimensional integrated intensity of the diffraction spot in the diffraction frame.

If a crystal is very thin, or contains planar disorder, for example stacking faults and/or twinning, the reflections will be elongated in reciprocal space. In extreme cases, the reflections may even be connected by streaking. In such cases the program will have problems identifying (separating) reflections from the diffraction spots. It is possible to manually pick and exclude the streaking in the program when reciprocal space is visualized, leaving only the diffraction spots close to the centres of the reflections. The merged reflections may not have accurate intensities for complete structure solution in the case of disorder but are usually at least sufficient for unit-cell determination in the following steps. The streaks and diffuse intensity are useful for studying disorder or short-range ordering in crystals. We are currently working on ways of processing streaks and diffuse scattering generated by disorder.

### 3.8. Unit-cell determination   

In this step the program looks for three reciprocal basis vectors that can be used to index all reflections. In *RED*, a method similar to that of the density-based clustering algorithm *DBSCAN* is adopted (Schlitt *et al.*, 2012 ▶). The reciprocal basis vectors are found by calculating the position difference vectors for the merged reflections and identifying three shortest noncoplanar difference vectors. Owing to individual errors in the positions of the reflections, the position difference vectors will appear not as one converged point but as isolated clouds when visualized, with each point in the cloud corresponding to one difference vector. The three shortest noncoplanar difference vectors are averaged within each cloud, to obtain the final reciprocal basis vectors. Unit cells defined by these basis vectors are reduced unit cells that may not reflect the highest symmetry of the crystal. The *RED* program provides options to transform the unit cell, using a user-defined orientation matrix. The function for identifying higher symmetries from the reduced unit cells is readily available in various crystallographic computer programs, for example, the *Computational Crystallography Toolbox* (Grosse-Kunstleve *et al.*, 2002 ▶; http://cctbx.sourceforge.net/).

### 3.9. Indexing   

Indexing of the reflections can be done in principle by decomposing the position vectors of the reflections over the reciprocal basis vectors. The resultant components give the indices *h*, *k* and *l* of the reflections. In experimental data, it is very common that reflections do not strictly follow the lattice defined by the basis vectors owing to errors in the reconstructed positions. For example, the lattice may appear to be bent, usually more at high-resolution regions. As a result, indices of the reflections will be decimal numbers rather than integers. The *RED* program uses a user-defined threshold to keep only reflections with indices within that threshold, rejecting reflections that deviate too much from the lattice. The default threshold value is ±0.1 in any of the indices *h*, *k* and *l*. It is possible to set different threshold values for *h*, *k* and *l*, respectively, if the unit-cell lengths differ a lot. Reflections with *hkl* indices deviating by more than the threshold value from integer values may be noise peaks in between reflections. Such reflections are excluded from the final list of reflections. The final indices of the remaining reflections are rounded to integers (see Table 1 ▶). It is possible to preserve the non-integer indices of reflections for incommensurately modulated structures and even quasi-crystals.

In order to take into account the bending of the reflection lattice, due to errors in the determination of the tilt axis orientation or distortions from the lens and camera, an adaptive indexing algorithm is adopted. The indexing starts from low-resolution reflections, which usually have only small deviations in positions from the lattice defined by the basis vectors. As the indexing goes out to higher-resolution reflections, it uses indexed reflections neighbouring the reflection being indexed as new position origins instead of the origin of the reciprocal lattice. As bending is usually small locally, this indexing scheme is tolerant to relatively large bending, including a spiral distortion seen at high resolution. In our experience, the indexing of reflections by the *RED* software has always been correct, even for large unit-cell dimensions and high indices.

### 3.10. Data and HKL list output   

After indexing, indices and intensities of the reflections are output to an HKL file in a standard HKLF4 format for *SHELX* (Sheldrick, 2008 ▶). This file can be used as an input to standard X-ray structure solution and refinement programs. Rich information about the reflections, including indices with decimals and after rounding, intensities, resolution, the number of ED frame where the intensity reaches maximum, and the coordinates of the reflection in the ED frame, are also given in the program, as shown in Table 1 ▶.

## 4. Application of *RED* to silicalite-1 and discussion   

Here we show an example of applying *RED* data collection and processing to silicalite-1. Calcined silicalite-1 at ambient conditions is monoclinic with space group *P*2_1_/*n* and unit cell *a* = 19.879, *b* = 20.107, *c* = 13.369 Å and β = 90.67°, with 24 symmetry-independent Si and 48 symmetry-independent O positions (Vankoningsveld *et al.*, 1990 ▶). Silicalite-1 often grows as twins and is one of the most complex zeolites solved by single-crystal X-ray diffraction. Calcined silicalite-1 crystals were dispersed in absolute ethanol and treated by ultrasonification for 2 min. A droplet of the suspension was transferred onto a carbon-coated copper grid and dried in air before the transmission electron microscope observation.

**Figure 10:**
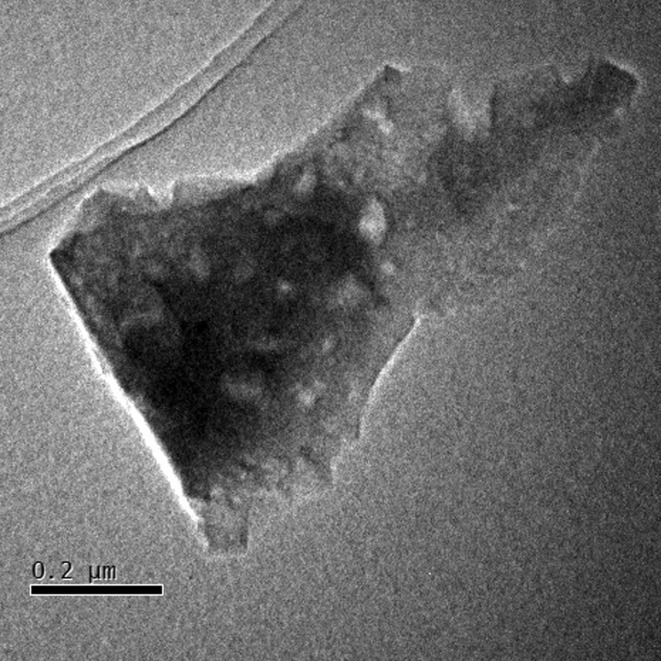
Low-magnification TEM image of the calcined silicalite-1 crystal used here for demonstrating the *RED* data collection and processing software.

**Figure 11:**
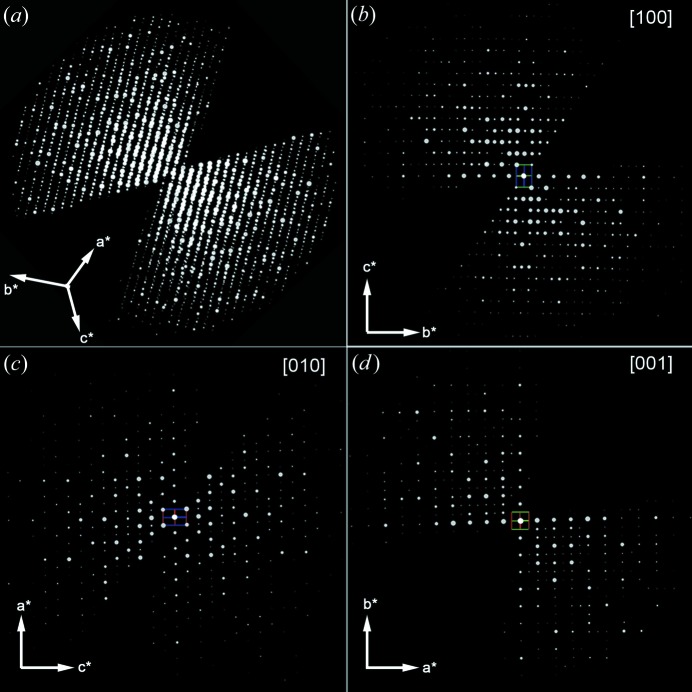
(*a*) The three-dimensional reciprocal lattice of calcined silicalite-1 reconstructed from the RED data and (*b*)–(*d*) two-dimensional slices cut from the reconstructed three-dimensional reciprocal lattice showing the (*b*) 0*kl*, (*c*) *h*0*l* and (*c*) *hk*0 planes.

The collection of three-dimensional RED data was controlled by the *RED* data collection program on a JEOL JEM2100 LaB_6_ transmission electron microscope at 200 kV. ED frames were recorded by a 12 bit Gatan ES500W Erlangshen camera side-mounted at a 35 mm port. The tilt axis of the electron beam was set in the same orientation as the tilt axis of the goniometer and stored by the *RED* software. A crystal of 0.8 × 0.4 × 0.2 µm in size (Fig. 10 ▶) was selected using a selected-area aperture. The crystal size was estimated from images taken at different goniometer tilt angles. The crystal height was adjusted to the eucentric height to minimize the shift of the crystal during the crystal tilt. The step of the beam tilt was 0.10° and the step of the goniometer was 2.0°. We suggest that the beam tilt range is slightly larger than the stage tilt to cover possible missing angles. The beam tilt range was ±1.10°, allowing a few overlapping ED frames at each crystal tilt. The exposure time for each ED frame was 0.5 s. The crystal can have arbitrary orientation and there was no need for any alignment of the crystal orientation. The position of the crystal was monitored after each crystal tilt and adjusted manually when necessary. The entire RED data set, with 1472 ED frames, was collected in 56 min, covering a tilt range from −65.3 to +64.2° using a single-tilt tomography holder. The data processing was performed using the *RED* data processing program, including peak search, intensity extraction, unit-cell determination and indexing of reflections. The reconstructed three-dimensional reciprocal lattice of silicalite-1 and the slices of the main zone axes are shown in Fig. 11 ▶.

The structure of silicalite-1 was solved from the maximal values of the ‘pixel’ intensities from this three-dimensional RED data set with direct methods by *SIR2011* (Burla *et al.*, 2012 ▶). All the Si and O atoms were found at reasonable positions. Final structure refinement was done using the *SHELXL97* program (Sheldrick, 2008 ▶) by minimizing the sum of squared deviations of *F* ^2^ using a full-matrix technique. All the Si and O atoms were refined isotropically. Detailed studies of the structure determination of calcined silicalite-1 using the RED method, including the effects of tilt range, tilt step and resolution on the determination of unit-cell parameters, and structure solution and refinement, are presented in a separate article (Su *et al.*, 2013 ▶). Crystallographic data and details of structure refinement using the RED data are summarized in Table 2 ▶.

A typical data collection involves taking about 1400 ED frames (in the range from −70 to +70°) at 0.10° beam tilt steps. With an exposure time of 0.5 s per frame and manual adjustment of the crystal shifts every 2°, the whole data collection takes about 60 min. The data processing takes 0.5−3 h. The resulting list with *hkl* indices and intensities is then used as input to standard programs developed for solving crystal structures from single-crystal X-ray data, such as *SHELX*, *SIR* or *SuperFlip* (Palatinus & Chapuis, 2007 ▶). In most cases, the correct crystal structure is found within minutes. Thus RED brings down the time taken for the whole procedure of collecting data and solving a crystal by single-crystal electron diffraction from weeks or months to a few hours.

Both ADT and RED provide data of a quality that is sufficient for routinely solving crystal structures of complexities up to around 100 unique atoms. Most structures that have been solved by three-dimensional electron diffraction so far have been inorganic (Jiang *et al.*, 2011 ▶; Martínez-Franco *et al.*, 2013 ▶), because of radiation damage which is more severe for organic molecules. However, some organic or inorganic–organic hybrid structures (Kolb *et al.*, 2010 ▶; Gorelik *et al.*, 2012 ▶; Bellussi *et al.*, 2012 ▶; Zhang *et al.*, 2013 ▶) and metal–organic frameworks (Feyand *et al.*, 2012 ▶; Willhammar *et al.*, 2013 ▶) have also been solved.

Apart from the problem of radiation damage, electron crystallography is still suffering from lower quality of the raw diffraction data, as compared to X-rays and neutrons. While electron diffraction often exceeds 1.0 Å resolution (sometimes reaching 0.5 Å), the intensities of symmetry-related reflections differ far more for electron diffraction than for X-ray diffraction, as seen in Table 2 ▶. This is an important issue and work is now being performed to find out the reasons for this problem, in order to develop procedures to correct for various factors that distort the diffraction intensities. We believe that the simpler geometry of the rotation method will facilitate such work.

## 5. Conclusions   

We present the *RED* data collection program, which controls the transmission electron microscope and the camera for automated three-dimensional electron diffraction data collection. Fine beam tilts are combined with coarse goniometer tilts to allow the Ewald sphere to sample three-dimensional reciprocal space. More than 1000 ED frames can be collected within one hour in either SAED mode or nano-diffraction mode. We also present the *RED* data processing program, which processes the ED frames generated from the *RED* data collection program or by other methods, for example ADT. The three-dimensional reciprocal lattice can be reconstructed and visualized by the *RED* software. The unit-cell parameters can be determined and the diffraction spots can be indexed. An output of list of reflections together with their *d* values and intensities is provided and can be used for *ab initio* structure determination of unknown crystals. The software is designed for a wide user community and aimed to be simple, user friendly and easy to learn.

Electron crystallography has finally reached a level of maturity, at a level similar to that of X-ray and neutron crystallography. Crystals that are too small for single-crystal X-ray diffraction and too complex for powder diffraction can be routinely solved using the RED method. The RED method is especially powerful for studying samples that contain several complex phases (including unknown new phases), which are difficult for powder diffraction. Individual powder grains with sizes down to tens of nanometres are seen as single crystals by electrons, albeit too small for single-crystal X-ray diffraction. We believe that the RED and ADT methods will mark a new era in crystallography.

## Supplementary Material

Technical details of software implementation and graphical user interfaces of the RED data collection program. DOI: 10.1107/S0021889813027714/nb5079sup1.pdf

## Figures and Tables

**Table 1:** An excerpt of the reflection table from the silicalite-1 data set given by the *RED* data processing software The table includes the indices with decimals (*h. k. l.*) and after rounding (*hkl*), the intensity (*I*), the *d* spacing, the ED frame number (Frame No.) where the intensity reaches maximum, and the coordinates of the reflection (*x*, *y*) in the ED frame. The complete list contains all reflections with *d* spacing 0.83. Note that Friedel pairs of reflections (*hkl* and *h* *k* *l*) appear just a few frames apart and have very similar intensities.

*h.*	*k.*	*l.*	*h*	*k*	*l*	*I*	*d* ()	Frame No.	*x*	*y*
0.97	0.95	0.01	1	1	0	2733.5	11.42	931	601	448
0.99	0.99	0.03	1	1	0	2727.3	10.97	932	679	475
0.04	1.01	0.97	0	1	1	2636.0	11.32	985	603	479
0.02	1.00	1.01	0	1	1	2657.4	10.86	985	679	442
0.03	1.02	1.01	0	1	1	1368.8	11.26	400	604	440
0.00	0.97	1.01	0	1	1	1194.0	11.07	401	675	485
1.03	1.00	0.03	1	1	0	311.1	11.14	30	598	468
1.03	0.97	0.02	1	1	0	335.7	10.96	32	682	460
1.96	0.01	0.00	2	0	0	1938.3	10.27	1403	668	496
1.98	0.01	0.01	2	0	0	1852.0	10.10	1404	621	416
0.02	0.03	1.96	0	0	2	548.9	10.25	148	644	508
0.02	0.00	2.00	0	0	2	519.3	9.87	146	635	416
0.98	0.99	1.01	1	1	1	1216.3	9.84	711	676	493
0.99	0.99	1.00	1	1	1	1161.6	9.82	710	606	429
1.00	0.97	0.98	1	1	1	475.9	9.83	75	682	482
1.01	0.99	1.00	1	1	1	408.2	9.68	73	594	444
1.00	1.00	1.01	1	1	1	1171.5	9.82	1225	598	457
1.00	1.00	1.01	1	1	1	1389.1	9.61	1228	695	457
1.99	0.01	1.02	2	0	1	227.2	9.07	1142	657	508
1.97	0.00	1.01	2	0	1	237.2	9.03	1141	636	407

**Table 2:** Crystallographic data of calcined silicalite-1 and details of structure refinement using the RED data

Chemical formula	Si_24_O_48_
Formula weight	1442.16
Temperature (K)	298
Wavelength ()	0.0251
Crystal system	Monoclinic
Space group	*P*2_1_/*n*
Unit cell (, )	*a* = 20.02, *b* = 20.25, *c* = 13.35, = 90.13, = 90.74, = 90.03
Volume (^3^)	5411
*Z*	4
Density (calculated) (Mg m^3^)	1.769
*F*(000)	940
Crystal size (m)†	0.8 0.4 0.2
Crystal colour	Colourless
Tilt range ()	65.3 to 64.2
Tilt step ()	0.10
No. of frames	1472
Resolution ()	1.05
Completeness	0.968
Reflections collected	13221
Unique reflections	4870
Unique reflections [*I* > 2(*I*)]‡	1833
Refined parameters	290
*R*_int_(*I*)	0.338
Final *R*(*I*) indices [*I* > 2(*I*)]‡	*R*_1_ = 0.318, *wR*_2_ = 0.571
*R*(*I*) indices (all data)	*R*_1_ = 0.408, *wR*_2_ = 0.633

† The crystal size was estimated from the TEM images taken at different goniometer tilt angles.

‡ (*I*) was calculated as the square root of the intensity *I*.

## Footnotes

1 Supporting information for this paper is available from the IUCr electronic archives (Reference: NB5079). Services for accessing this material are described at the back of the journal.

## Appendix A Calibration of the beam tilt and image shift deflectors

The *RED* data collection program uses beam tilt deflectors to tilt the electron beam and two image shift (IS) deflectors, IS1 and IS2, to bring the image and diffraction pattern back. To ensure that the images and diffraction patterns are nearly stationary when beam tilt is applied, it is important to apply the correct changes to the electric currents of IS1 and IS2. Therefore, the effects of the different deflectors in terms of image shift and diffraction pattern shift, *i.e*. the relation between the amount of change in current and the amount of image and diffraction shift, has to be determined. This is done in two calibration processes, one in diffraction mode and one in image mode. In diffraction mode, the currents of the beam tilt deflectors and two image shift deflectors are changed, one at a time, and the corresponding shift of the diffraction pattern is registered. In image mode, the currents of the two image shift deflectors are changed one at a time and the corresponding shift of the image is registered. The beam tilt deflectors are not tested in image mode as beam tilt does not introduce image shift after the transmission electron microscope is properly aligned. The ratios between the change of electric currents and shifts (diffraction patterns and images) are saved and will be used in the de-scan (Fig. S1b). Each deflector has *x* and *y* components; thus the change of current can be written as a vector:

Image or diffraction shift can also be written as a vector containing shifts in the **x** and **y** directions:

The change of electric currents in the beam tilt and IS1 and IS2 deflectors, and the corresponding diffraction shift, can be related by a 2 × 2 matrix *M*:

where the subscript BT stands for beam tilt. The change of electric currents in the deflectors and the corresponding image shift can be related in a similar way:

For image de-scan, the ratio between  and  will be set such that the overall image shift is zero:

The ratio can also be expressed as a matrix:

where

After calibration, four matrices, , ,  and , are determined and later used in the de-scan of beam tilt.

For diffraction scan, a combination of  and  needs to be found for a given  in order to keep the overall shift of the diffraction patterns zero:

Solution of the linear equations (7) and (9) gives  and .

In our experience, the transmission electron microscope is stable enough that these values only have to be determined once and stored in the computer. They can then be loaded for each RED data collection and used for a long period of time (several months).
