# Supplementary material for: Three-dimensional rotation electron diffraction: software RED for automated data collection and data processing
Source: J Appl Crystallogr. 2013 Nov 15;46(Pt 6):1863–73. doi: 10.1107/S0021889813027714 (PMC3831301; doi:10.1107/S0021889813027714)
Supplement: Supplementary file 1 [file j-46-01863-sup1.pdf]

Supplementary information

Three-dimensional rotation electron diffraction: software RED for automated data collection and data processing

Wei Wan[a*], Junliang Sun[a*], Jie Su[a], Sven Hovmöller[a] and Xiaodong Zou[a*]

[a]Inorganic and Structural Chemistry and Berzelii Centre EXSELENT on Porous Materials, Department of Materials and Environmental Chemistry, Stockholm University, Arrhenius Laboratory, Stockholm, SE-106 91, Sweden

Correspondence emails: wei.wan@mmk.su.se; junliang.sun@mmk.su.se; xzou@mmk.su.se

Contents:

S1. Technical details of software implementation

S2. Graphical user interfaces of the data collection program

References

S1. Technical details of software implementation

The RED program package, consisting of the data collection and data processing programs, is written in C++. The graphical user interfaces are implemented using the *Qt framework*. The data collection program has been implemented for JEOL TEMs. It controls the microscope using the external TEM control which is a component object model (COM) module provided by JEOL. Reading data from the camera is implemented using a DigitalMicrograph plugin provided in the *Gatan software development kit*.

3D visualization of reciprocal space in the data processing program is performed using *OpenGL*. The program has optimum performance in visualization with a graphics card supporting hardware OpenGL acceleration. Fast Fourier transform calculations in cross correlation is done using the *AMD Core Math Library*. The data processing program has been tested on Windows and Linux. The data collection program works on Windows only, as it needs to interface with the camera and microscope software that run only on Windows.

S2. Graphical user interfaces of the data collection program

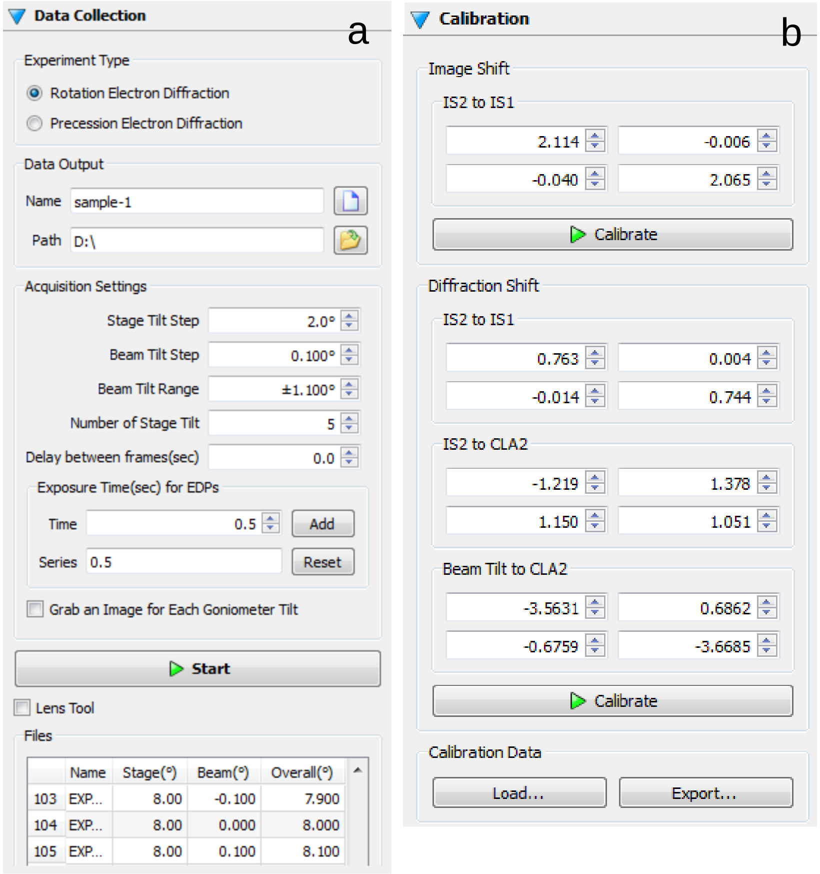

Figure S1 Data collection window (a) and calibration window (b) of the RED data collection program.

References

AMD Core Math Library, http://developer.amd.com/tools-and-sdks/cpu-development/amd-core-math-library-acml/

DigitalMicrograph software development kit, http://www.gatan.com/scripting/SDK.php

OpenGL, http://www.opengl.org/

Qt framework, http://qt-project.org/
